# Willows: a memory efficient tree and forest construction package

**DOI:** 10.1186/1471-2105-10-130

**Published:** 2009-05-05

**Authors:** Heping Zhang, Minghui Wang, Xiang Chen

**Affiliations:** 1 Department of Epidemiology and Public Health, Yale University School of Medicine, New Haven, CT 06520-8034, USA

## Abstract

**Background:**

Existing tree and forest methods are powerful bioinformatics tools to explore high dimensional data including high throughput genomic data. However, they cannot deal with the data generated by recent genotyping platforms for single nucleotide polymorphisms due to the massive size of the data and its excessive memory demand.

**Results:**

Using the recursive partitioning technique, we developed a new software package, Willows, to maximize the utility of the computer memory and make it feasible to analyze massive genotype data. This package includes three tree-based methods – classification tree, random forest, and deterministic forest, and can efficiently handle the massive amount of SNP data. In addition, this package can easily set different options (e.g., algorithms and specifications) and predict the class of test samples.

**Conclusion:**

We developed Willows in a user friendly interface with the goal of maximizing the use of memory, which is critical for analysis of genomic data. The Willows package is well documented and publicly available at .

## Background

Successes of genomewide association (GWA) studies have demonstrated repeatedly that single nucleotide polymorphisms (SNPs) can be used to identify genetic variants underlying complex diseases [1-5]. Thanks to those successes, GWA studies have emerged as the most effective study designs for identifying candidate genes.

Classification trees and forest-based methods [6-9] are powerful tools for identifying complex relationships between a response and many predictors, particularly if the predictors have interactive effects on the response. These methods have been widely used, such as in the analyses of genomic data [10-13]. However, the grand scale of the GWA data presents a significant computational challenge to any data analysis. For example, the genotype data from the Framingham Heart Study (FHS, 9,300 subjects and 550,000 SNPs) require more than 38.1 GB memory for input when each genotype at a SNP marker is stored in the double data type or 4.8 GB when stored in the byte type. For a typical GWA study, e.g., the Cancer Genetic Markers of Susceptibility (CGEMS) breast cancer projects (2,434 subjects and 550,000 SNPs) [14], the genotype data occupy 10 GB in the double type and 1.2 GB in the byte type. None of the existing tree/forest tools are capable of analyzing these massive data in commonly available computing facilities. It is noteworthy that PLINK [15] and Chen, *et al*. [16] already utilize efficient memory use algorithms similar to what we propose to use in trees and forests, and the compressed data format designed by PLINK has been adopted by NCBI to distribute GWA data. Thus, incorporating an efficient memory use algorithm in other statistical methods such as tree- and forest-based methods is imperative in order to apply those well-established methods for analyzing ultra-dense SNP data.

To this end, we have developed a new software package, Willows. The statistical method is based on the classical recursive partitioning technique [17,18]. Compression/decompression algorithms have been implemented in Willows to efficiently reduce the memory level used for the storage and analysis of SNP data. Three recursive partitioning-based methods – classification tree, random forest, and deterministic forest – have been included in this package, which can efficiently handle the massive amount of SNP data. In addition, this package is equipped with a user-friendly graphic interface by which users can easily select different options (e.g., algorithms and specifications) and predict the class of a test sample.

## Implementation

### Classification tree

Classification tree is based on recursive partitioning method [6,18]. It extracts homogeneous strata from the sample and builds a classification rule to predict class membership. A splitting rule consists of two components: a predictor and its corresponding threshold. The quality of a splitting rule is measured by node impurity such as Gini index or entropy. Once the root node is split into two daughter nodes, the daughter nodes can be further split by repeating the splitting procedure. This partitioning process continues recursively until no more split is possible. To avoid over fitting, pruning procedures is used to eliminate redundant nodes [18-20].

### Random forest

Random forests [7] grows many classification trees instead of one. Suppose that the sample size in a data set is N. First, we draw N observations at random from the original data with replacement. Then, we grow a tree using this bootstrap sample. Trees in a random forest are built differently from the classification tree described in the previous section in the following two ways: (a) the trees in the random forest are not pruned; and (b) we do not consider all predictors in selecting the optimal node-split. In fact, if there are M predictors in the original data set, m out of M predictors are chosen randomly to split a node; here m is a pre-specified, much smaller number than M.

Random forest ranks variables by a variable importance index [7], which reflects the "importance" of a variable on the basis of the classification accuracy, while considering the interaction among variables. Specifically, in a random forest each tree is constructed using a different cohort of bootstrap samples from the original cohort. About one-third of the samples are left out of the bootstrap samples and hence not used in the construction of the tree. These left-out samples are referred to as the out-of-bag (oob) samples. To determine the importance of a variable, first the values of the variable (i.e., predictor) in the oob samples are randomly permuted; then both the original oob samples and the permuted oob samples are classified by the corresponding tree. The difference in the correct classification rates between the original and permuted oob samples determines the importance of the variable, and the variable importance is obtained by averaging the differences over all trees in the random forest.

### Deterministic forest

Like a random forest, a deterministic forest [8,11] is also an ensemble of classification trees. Because of the large number of covariates, multiple splits may have very similar performance in terms of the quality of split and the prediction accuracy of the outcome. Thus, it is useful to consider all competitive splits, and construct a forest consisting of these competitive trees. Specifically, a pre-specified number (for example, 20) of the top splits of the root node and a pre-specified number (for example, 3) of the top splits of the two daughter nodes of the root node are selected. These combinations generate a total of 180 possible trees, leading to a deterministic forest. The frequency of each predictor being used to split a node is indicative of the importance of the predictor. A deterministic forest is different from a random forest in that it is constructed through a deterministic and reproducible manner and that the trees in the deterministic forest tend to be very limited in size. A deterministic forest is not only computationally more efficient than a random forest, but also its reproducibility makes it easier to interpret.

### Missing value

Considering the massive amount of SNPs, we expect some SNP genotypes may be missing either due to mishandling or poor quality. There are two simple approaches to dealing with missing SNPs. First, we can impute the missing SNP based on the allele frequency in the data or the haplotype block covering the missing SNP. After this imputation, all of the missing SNPs are replaced by the imputed SNPs and the "completed" data are then fed to Willows. Alternatively, the Missings Together Approach [18] can be adopted; namely, the subjects with missing SNPs are grouped together so that they can be easily tracked. In the tree framework, the first approach is expected to produce trees with a lower misclassification rate than the second approach. However, when forests are constructed, it warrants a further comparison as to which of two approaches leads to better performing forests.

### Compression Algorithms

In genetic studies, a SNP-based genotype has only four possible choices: AA, AB, BB or missing. Each choice can be represented by 2 bits. Thus, 16 genotypes can be packed into one integer data type (4 bytes) in Java or C++ using bit shift operators. The theoretical compression ratio is 4:1 compared to the byte storage scheme and 32:1 compared to the double storage scheme.

### Implementation

Willows, implemented in C and Java, comes with a user-friendly graphic user interface (GUI) on Windows, Linux and Mac OS X. It also can be executed from the command line on Windows, Linux and Mac OS X.

## Results and discussion

The performance of Willows was analyzed on a computer equipped with 2.33 GHz processor and 2 GB physical memory running on Microsoft Windows XP Professional Version.

### Simulated data

The compression and decompression operations for a specific genotype take a constant operation time using bit operators. In fact, the time required for these operations is negligible comparing to the overall running time. For example, we randomly generated two simulated data sets, which had 10, 000 SNPs and 100, 000 SNPs, respectively. Both data sets contained 1, 000 subjects. For each data set, we built classification trees, a random forest of 100 trees, and a deterministic forest of 8 trees, respectively, on a computer described above. The number of SNPs used to split at each node in the random forest is set to be int(log_2 _*M*) + 1, where M is the number of SNPs. The running time with the compressed and uncompressed operations is given in Table 1, and differs very little with or without the compressed operations.

**Table 1:** Run time (in seconds) of the operations.

	SNPs	Classification tree	Random forest	Deterministic forest
Compressed	10, 000	70	7	480
Uncompressed	10, 000	68	6	470
Compressed	100, 000	257	96	673
Uncompressed	100, 000	249	94	640

### CGEMS

For a typical GWA study, e.g., the Cancer Genetic Markers of Susceptibility (CGEMS) breast cancer projects [14], which contains 2,434 subjects and 550,000 SNPs, the genotype data occupy 10 GB in the double type and 1.2 GB in the byte type. As we did for the simulated data sets, we built classification trees, a random forest of 1000 trees, and a deterministic forest of 8 trees, respectively. The number of SNPs used to split on at each node in random forest is set to be int(log_2 _550000) + 1. Table 2 displays the time of using Willows to analyze CGEMS data, and it demonstrates that with the efficient use of memory, we can indeed construct classification trees and forests from typical GWA data.

**Table 2:** Computation time (in seconds) for analyzing the CEGM data set

Memory	Loading data	Classification trees	Random forest	Deterministic forest
0.32 Gb	1698	2562	485	12170

### Input files

Willows supports input files in a text format: the first line indicates the variable type (response, nominal or ordinal) with no particular order. Among various features is the prediction function that predicts the response class based on the predictors. Additional input files are necessary for this feature. We refer to the supplementary information on our website.

### Output results

**Figure 1:**
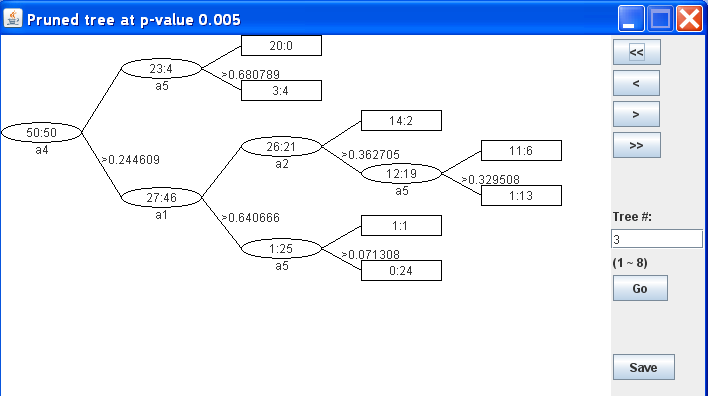
**Tree structure**.

The main output produced by Willows is the tree structures. An example is provided in Figure 1. In this figure, internal and terminal nodes are represented by ellipsoids and rectangles, respectively. The frequency counts of the outcome are displayed inside each node, and the splitting variable and the corresponding thresholds are provided for internal nodes.

**Figure 2:**
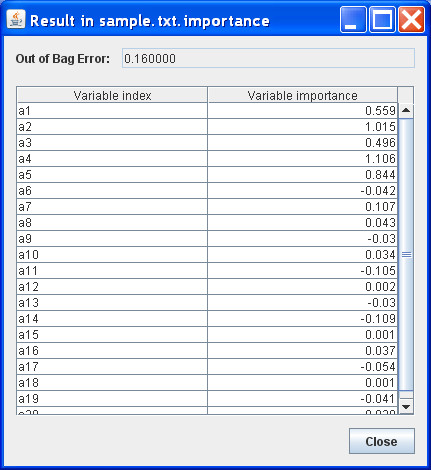
**Importance score results in the random forest**.

**Figure 3:**
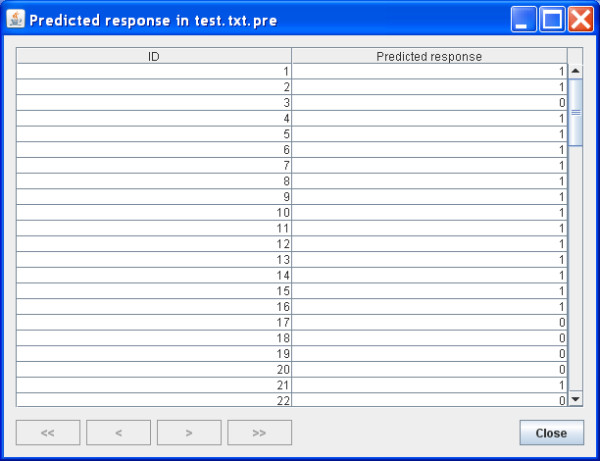
**Prediction results in a test sample**.

Depending on the needs, other outputs including the importance score of each variable and the predicted classes in a test sample can be viewed. For example, Figure 2 and Figure 3 show the importance score and prediction results of the two simulated data sets. Furthermore, all of the results are saved in local files for future view. Detailed instructions are provided in our website.

## Conclusion

GWA studies have produced landmark successes in identifying genetic variants for complex diseases. Due to the large size of the data generated from GWA studies, data management and analysis has been a major hurtle to overcome for GWA studies. One of the immediate challenges is the memory management for GWA databases, especially for prevailing 32-bit operation systems. Parallel supercomputers are useful to accelerate the computation when the computational tasks are "parallel," but this may not be the case or may be challenging to implement in GWA studies. Furthermore, parallel supercomputers are not easily accessible, and even if they are available, data confidentiality and security restrictions may not allow the transfer of the genomic data to a networked supercomputer, as those released by dbGap . Thus, it is ideal to have more accessible and efficient computing software. In fact, some of the dbGap data sets have been distributed in a compressed binary format designed in PLINK and incompatible for other statistical software including trees and forests. To this end, Willows implements three classifiers in a user friendly interface with the goal of maximizing the use of memory, which is necessary for analysis of GWA SNP data.

## Availability and requirements

• **Project name**: Willows

• **Project home page**: .

• **Operating system(s)**: Multiple platform (tested on Windows, Linux and Mac OS X).

• **Programming language**: C++ and Java.

• **Other requirements**: Java 1.6+.

• **License**: Free for non-commercial use.

## Authors' contributions

All authors jointly developed the methods and wrote the article. They read and approved the final manuscript.

## References

[B1] Helgadottir A, Thorleifsson G, Manolescu A, Gretarsdottir S, Blondal T, Jonasdottir A, Jonasdottir A, Sigurdsson A, Baker A, Palsson A (2007). A common variant on chromosome 9p21 affects the risk of myocardial infarction. Science.

[B2] Klein RJ, Zeiss C, Chew EY, Tsai JY, Sackler RS, Haynes C, Henning AK, SanGiovanni JP, Mane SM, Mayne ST (2005). Complement factor H polymorphism in age-related macular degeneration. Science.

[B3] McPherson R, Pertsemlidis A, Kavaslar N, Stewart A, Roberts R, Cox DR, Hinds DA, Pennacchio LA, Tybjaerg-Hansen A, Folsom AR (2007). A common allele on chromosome 9 associated with coronary heart disease. Science.

[B4] Samani NJ, Erdmann J, Hall AS, Hengstenberg C, Mangino M, Mayer B, Dixon RJ, Meitinger T, Braund P, Wichmann HE (2007). Genomewide association analysis of coronary artery disease. N Engl J Med.

[B5] Consortium TWTCC (2007). Genome-wide association study of 14,000 cases of seven common diseases and 3,000 shared controls. Nature.

[B6] Breiman L, Friedman F, Stone C, Olshen R (1984). Classification and regression trees.

[B7] Breiman L (2001). Random Forests. Machine Learning.

[B8] Zhang H, Yu C-Y, Singer B (2003). Cell and tumor classification using gene expression data: Construction of forests. Proc Natl Acad Sci USA.

[B9] Zhang H, Ye Y (2008). A tree-based method for modeling a multivariate ordinal response. Stat Interface.

[B10] Zhang H, Bonney G (2000). Use of classification trees for association studies. Genet Epidemiol.

[B11] Ye Y, Zhong X, Zhang H (2005). A genome-wide tree- and forest-based association analysis of comorbidity of alcoholism and smoking. BMC Genet.

[B12] Chen X, Liu CT, Zhang M, Zhang H (2007). A forest-based approach to identifying gene and gene gene interactions. Proc Natl Acad Sci USA.

[B13] Bureau A, Dupuis J, Falls K, Lunetta KL, Hayward B, Keith TP, Van Eerdewegh P (2005). Identifying SNPs predictive of phenotype using random forests. Genet Epidemiol.

[B14] Hunter DJ, Kraft P, Jacobs KB, Cox DG, Yeager M, Hankinson SE, Wacholder S, Wang Z, Welch R, Hutchinson A (2007). A genome-wide association study identifies alleles in FGFR2 associated with risk of sporadic postmenopausal breast cancer. Nat Genet.

[B15] Purcell S, Neale B, Todd-Brown K, Thomas L, Ferreira MA, Bender D, Maller J, Sklar P, de Bakker PI, Daly MJ (2007). PLINK: a tool set for whole-genome association and population-based linkage analyses. Am J Hum Genet.

[B16] Chen X, Zhang M, Wang M, Zhu W, Cho K, Zhang H (2009). Memory management in genomewide association studies. BMC Proc.

[B17] Breiman L (1984). Classification and regression trees.

[B18] Zhang H, Singer B (1999). Recursive partitioning in the health sciences.

[B19] Zhang H, Bracken MB (1995). Tree-based risk factor analysis of preterm delivery and small-for-gestational-age birth. Am J Epidemiol.

[B20] Zhang H, Holford T, Bracken MB (1996). A tree-based method of analysis for prospective studies. Stat Med.

